# Inflammatory Pseudotumor of the Temporal Bone: Three Cases and a Review of the Literature

**DOI:** 10.1155/2013/480476

**Published:** 2013-01-09

**Authors:** Huiqin Tian, Tingting Liu, Cong Wang, Lijun Tang, Zhibin Chen, Guangqian Xing

**Affiliations:** ^1^Department of Otolaryngology, First Affiliated Hospital of Nanjing Medical University, 300 Guangzhou Road, Nanjing 210029, China; ^2^Department of Pathology, First Affiliated Hospital of Nanjing Medical University, Nanjing 210029, China; ^3^Department of Radiology, First Affiliated Hospital of Nanjing Medical University, Nanjing 210029, China

## Abstract

Inflammatory pseudotumor (IP) is a clinically aggressive but histologically benign condition of unknown cause. Its appearance in the temporal bone is uncommon. We present clinical, radiological, and histopathologic findings of three cases originating in the temporal bone. In the first case, a simultaneous IP of the temporal bone and parotid gland was found with histopathologic confirmation. In the second case, an enlarged cervical node, which was also believed to be related to IP, was observed accompanied with the temporal lesion. While the third case presented with chronic suppurative otitis media. Two of them were treated by surgery alone with complete resolve of the diseases. Another one underwent tympanomastoidectomy in combination with oral steroids, radiation, and chemotherapy, but the IP still recurred. A comprehensive review of the literature on clinical features of the temporal pseudotumor was conducted.

## 1. Introduction

Inflammatory pseudotumor (IP), first described by Brich-Hirshfield in 1905 and named by Umiker et al. in 1954, is a clinically invasive, yet histologically benign mass lesion of unknown etiology. It has a variety of histologic presentations and has been recognized under various names like plasma cell granuloma, inflammatory myofibroblastic tumor, histiocytoma complex, xanthomatous pseudotumor, fibrous xanthoma, and inflammatory myofibrohistiocytic proliferation [1]. IP most commonly arises in the lung. Other sites include abdomen, retroperitoneum, or pelvis, upper respiratory tract, trunk, and extremities in descending order. Less than 5% of it occurs in the head and neck with orbit the most frequently involved site [1]. 

IP of the temporal bone is uncommon but remains important given its recurrent and destructive natures. Only a limited number of these cases have previously been reported [2–24]. Here, we present three new cases with variable manifestations. A comprehensive review of the English-speaking literature on clinical features of the temporal pseudotumor was performed for comparison.

## 2. Case Report

**Case 1**

**Figure 1:**
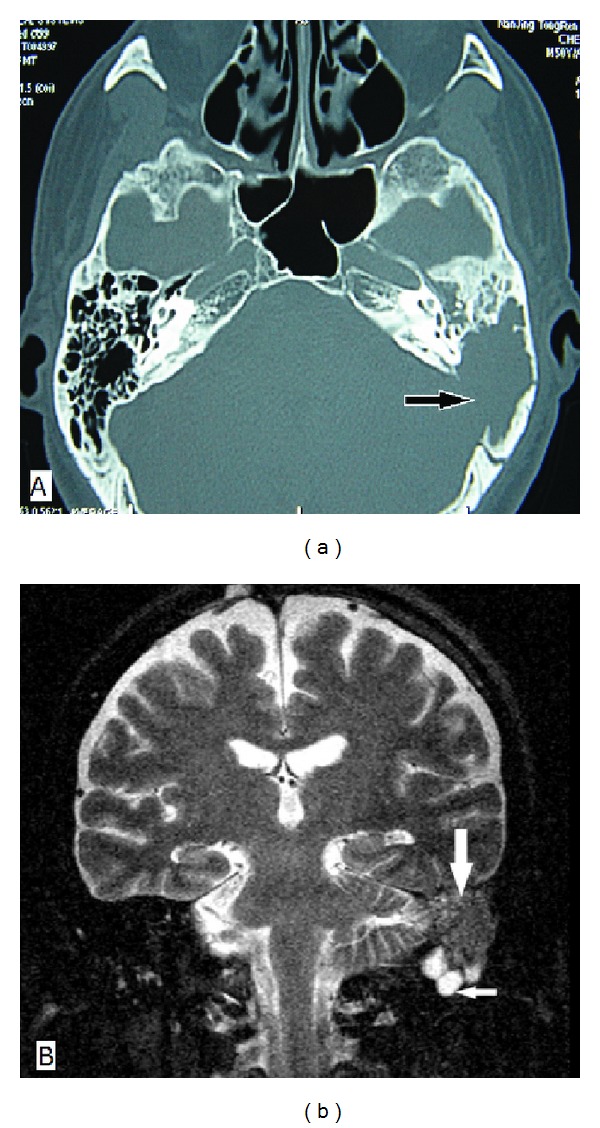
Imaging findings of Case 1. (a) Axial CT of the temporal bone shows a soft-tissue mass in the left middle ear and mastoid cavity with extension into the sigmoid sinus plate (arrow); (b) coronal MRI shows a slightly low-signal-intensity mass on T2-weighted images in the left mastoid (large arrow). Several inferior lymph nodes are seen (small arrow).

**Figure 2:**
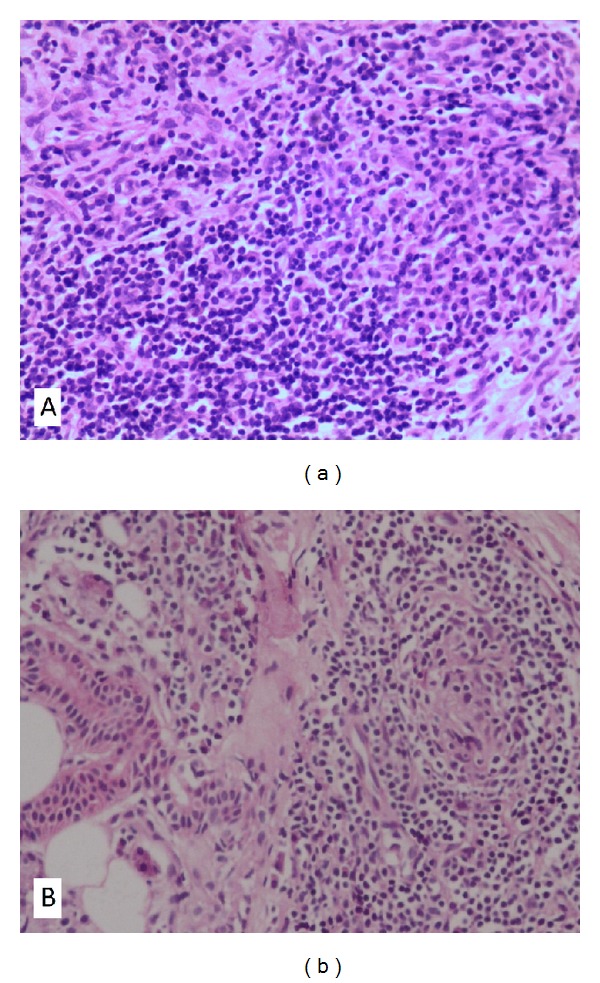
Histological pictures of Case 1. (a) Specimen obtained from the temporal bone demonstrates fibroblasts proliferation in an inflammatory infiltration background with predominance of plasma cells (H&E; original magnification ×200); (b) specimen of the parotid gland shows proliferation of lymphocytes, and plasma cells. A granuloma-like structure composed of fibroblasts, lymphocytes, and plasma cells was seen (H&E; original magnification ×200).

In June 2008, an otherwise healthy 51-year-old man presented with left-sided ear fullness and hearing loss for a few days. He was diagnosed as otitis media with effusion and underwent a tympanocentesis plus oral antibiotics and steroids. The symptoms alleviated but returned with aggravated hearing and a new onset of otalgia one month later. Otoscopic and audiological examinations demonstrated intact tympanic membrane and severe mixed hearing loss of the involved ear. A computed tomographic (CT) scan revealed an erosive soft-tissue mass of the left temporal bone extending into the sigmoid sinus (Figure 1(a)). Magnetic resonance imaging (MRI) showed a slightly low-signal-intensity lesion on T2-weighted images at the same location and several ipsilateral cervical nodes (Figure 1(b)). A mastoidectomy was performed. During this procedure, inflamed tissue was found filling the mastoid cavity and eroding into sigmoid sinus plate and the surrounding dura. Postoperative pathology was consistent with IP (Figure 2(a)), and immunohistochemical staining for *κ* and *λ* light-chains exhibited a polyclonal origin of plasma cells. A differential diagnosis was also made which excluded the possibility of neoplastic or granulomatous etiologies (plasmacytoma, Langenhans cell histiocytosis). During the patient's admission, he was noticed a swelling on his left parotid area and several enlarged lymph nodes in the upper neck. They all shrunk dramatically after tympanomastoidectomy without any other treatments.

During the 4-year follow-up period, the IP of the patient experienced three recurrences. The first recurrence occurred 6 months after mastoidectomy, when he complained of progression of left parotid and neck swellings and underwent partial parotidectomy with lymphadenectomy at another hospital. The postoperative histology revealed that the inflammatory infiltrate of the parotid specimen was composed of fibroblasts, lymphocytes, and plasma cells (Figure 2(b)). The second recurrence came one month after the parotidectomy. The patient reported left otalgia with new onsets of left-sided exophthalmos, orbital pain, diplopia, and headache. CT scan showed no gross mass in the orbit, but MRI revealed enhancing lesions in the left cerebellopontine area, cavernous sinus, temporal lobe meninges, and a suspicious involvement of cranial nerve. He was then commenced on a reducing regime of oral prednisone for 4 months accompanied by cyclophosphamide chemotherapy and radiotherapy. As we had expected, his otalgia, exophthalmos, diplopia, orbital pain, and headache were completely resolved. Thereafter, the patient was well until he experienced a relapse of exophthalmos 16 months later (the third recurrence). A tapering steroid was prescribed again which subsequently relieved his symptom. Now the patient is still under followup and remains symptom free.

**Case 2**

**Figure 3:**
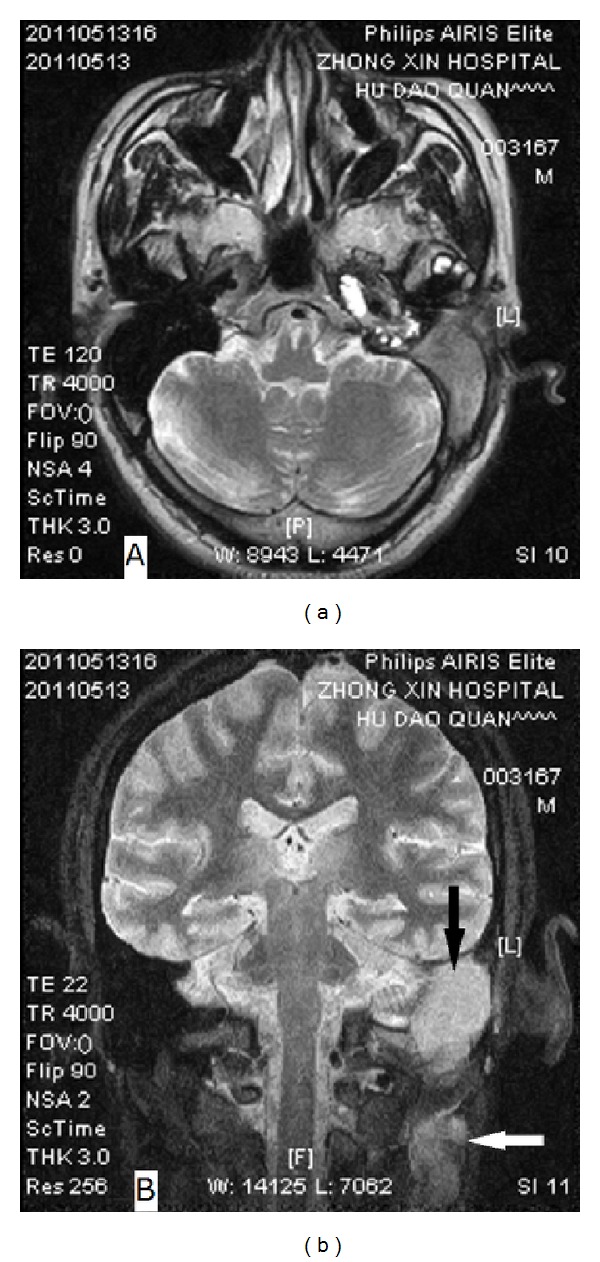
MR images of Case 2. (a) T2-weighted axial image shows a hypointensity lesion in the left mastoid; (b) PDW coronal image demonstrates two nonhomogenous and slightly high-signal-intensity mass in the left mastoid (black arrow) and ipsilateral neck (white arrow), respectively.

**Figure 4:**
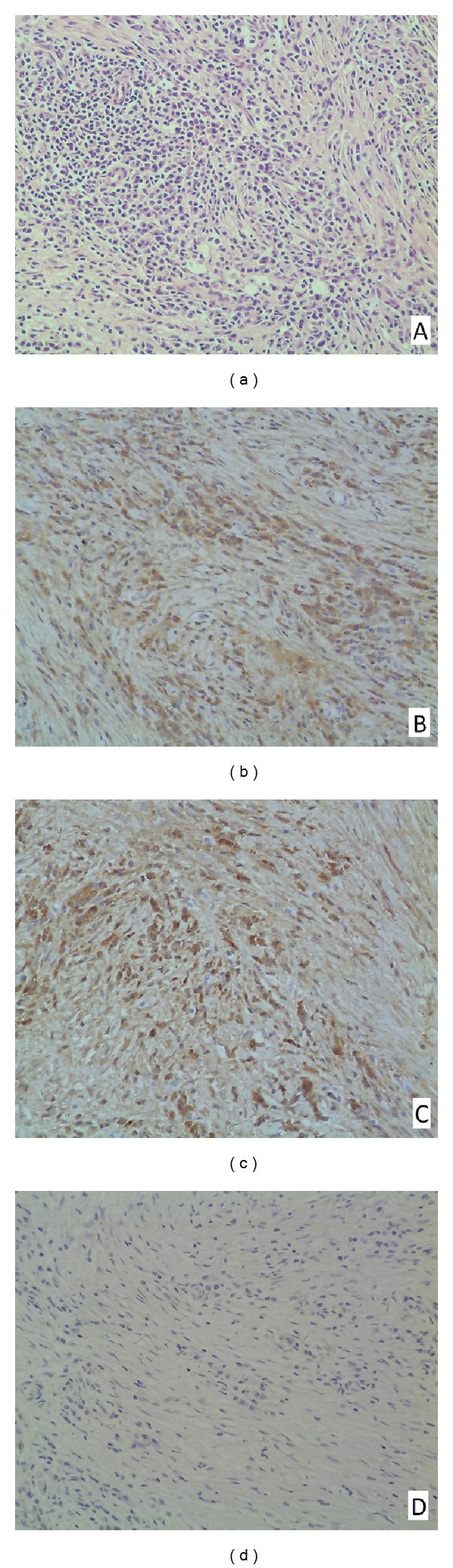
Histopathologic feature (a) and immunohistochemical staining (b–d) of Case 2. (a) Lymphoplasmacytic infiltration with predominance of plasma cells and proliferation of myofibroblasts (H&E; original magnification ×200); (b) positive reaction of plasma cells for IgG *κ* (original magnification ×200); (c) positive reaction of plasma cells for IgG *λ* (original magnification ×200); and (d) negative reaction of spindle cells for smooth muscle actin (original magnification ×200).

The patient is a 44-year-old man who presented with intermittent left-sided otorrhea, otalgia, tinnitus, decreased hearing, and fluctuant neck swelling for 3 months. His medical history was unremarkable except for head trauma (fracture of the left temporal bone) one year prior to presentation. On physical examination, his left eardrum was intact but thickened, and a smooth cervical mass (enlarged lymph node) measuring 3 cm × 5 cm was palpable on the left. Audiological studies exhibited moderately mixed hearing loss of the involved ear. Temporal bone CT revealed a soft-tissue mass in the left middle ear and mastoid cavity with bony erosion. MRI showed two nonhomogenous mass lesions in the left temporal bone and ipsilateral neck that was hypointensity on T2-weighted images (Figure 3(a)) and slightly high intensity on proton density weighted (PDW) images (Figure 3(b)), respectively. Complete blood cell count, blood chemistry, urinalysis, chest X-ray, and serologic test for syphilis, EBV, CMV, and HIV were generally normal. The patient was then planned for a tympanomastoidectomy both for diagnosis and treatment. Intraoperatively, the middle ear and mastoid cavity were found filling with the pinkish neoplasm which encased the vertical segment of the facial nerve. The surgical margin between the neoplasm and normal tissues was vague. Extensive bony erosion was seen in the plates of sigmoid sinus, superior petrosal sinus, tegmen tympani, and posterior cranial fossa. Postoperative histopathology of the surgical specimen revealed characteristic of IP (Figure 4(a)). Further immunohistochemical staining exhibited CD38 (++), CD3 (+), CD20 (+), CK (+), *κ* (+), *λ* (+), and SMA (−) (Figures 4(b), 4(c), and 4(d)). Similar to that of the first case, the neck mass in this patient disappeared soon after the mastoid surgery. During our 14-month followup, the patient was well although additional treatment was not performed.

**Case 3**

**Figure 5:**
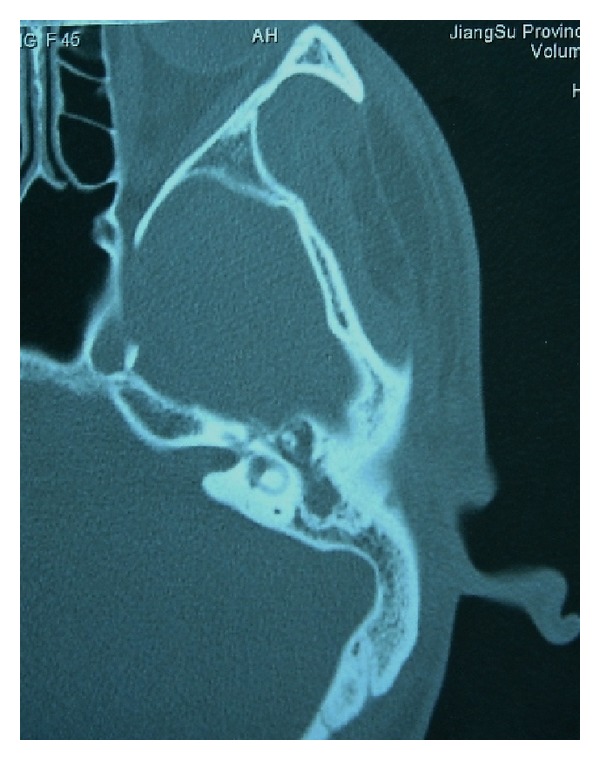
Axial CT of the temporal bone in Case 3.

A 50-year-old male was admitted to our hospital with a 7-year history of intermittent otorrhea and decreased hearing in the left ear. He denied tinnitus or vertigo. His medical history was otherwise unremarkable. Otoscopic and audiological examinations revealed a small perforation at the flaccid part of tympanic membrane and a moderate conductive hearing loss on the left. CT scans of the temporal bone showed opacification of epitympanum and mastoid antrum with erosion of malleal head and incus of the involved ear (Figure 5). With the initial diagnosis of chronic suppurative otitis media, a canal-wall down mastoidectomy with tympanoplasty was performed. Intraoperatively, it was found that the hyperplastic soft tissue filled both the mastoid antrum and epitympanum, which was removed completely without difficulty. There was no erosion of the tegmen, and the middle ear cavity was free of disease.

The postoperative course was uneventful. A histopathological exam showed proliferation of fibroblast with infiltration of numerous plasma cells and lymphocytes, and immunohistochemical staining for *κ* and *λ* light-chains also exhibited a polyclonal origin of plasma cells. Theses features were consistent with an IP. The patient's mastoid cavity healed well, and there was no evidence of recurrence for 11-month followup.

## 3. Literature Review

Pseudotumor of the temporal bone is an unusual condition. According to the literatures searched in the Pubmed database from 1970 to the present using the medical subject headings “inflammatory pseudotumor/plasma cell granuloma/inflammatory myofibroblastic tumor” and “temporal bone/middle ear/mastoid/facial nerve,” only 29 evaluable cases were identified (Table 1) [2–24]. There are seventeen men and twelve women, with an average age of 38.0 years (median age, 39.0 years; range, 2.5–75 years). The presenting symptoms are mainly hearing loss (20/29), otalgia (12/29), otorrhea (10/29), facial palsy (7/29), and dizziness/vertigo (5/29). Complains of tinnitus, ear fullness, headache, retrobulbar pain, diplopia, facial pain, and visual dimness are all uncommon. The mastoid and middle ear are the most frequent sites involved, representing 86.2%  (25/29) and 75.9% (22/29) of reported cases, respectively. However, temporal pseudotumor appears to have a more aggressive and unpredictable courses than IPs in other body sites. IPs occurring in the middle ear and mastoid may erode into the surrounding dura, sigmoid sinus, tentorium, and even brain parenchyma [7, 9–11, 15, 16]. Intratemporal extensions to the otic capsule, facial nerve, petrous apex, and internal auditory canal are common [6, 8, 10, 14, 21]. 

IPs is usually isolated. There is only one case had a concomitant IP of the temporal bone and lung [13], and three had indirect orbit involvement [4, 11, 13]. As for treatments, surgery is usually of the first priority, occurring in 24 reported cases (82.8%). The outcomes are some different among patients: two died of the disease progression soon after the diagnosis [8, 21], two experienced spontaneous improvement without any treatment [18, 22], and the others had subsequently complete or partial remission during the follow up period. Recurrent disease occurred in three of the twenty nine cases (10.3%), and all of them relapsed within a few months after treatment [6, 8, 19].

## 4. Discussion

IP most commonly involves the lung and orbit, but it has been described in almost any location, in both sexes, at all ages [1, 10]. It is characterized by the presence of a mass lesion that may mimic malignancy and that is composed of spindle cells mixed with variable amounts of extracellular collagen, lymphocytes, and plasma cells [25, 26]. The pathogenesis of IP is still obscure but immunological and infectious causes have been postulated [10, 27]. Recently, some IPs have been found to be associated with IgG4-related sclerosing diseases [28, 29], and Masterson et al. [28] recommended a full review of all histological specimens in patients with a diagnosis of IP or inflammatory myofibroblastic tumor. Consideration should be given to staining for IgG4, together with measurement of serum levels. In our first patient, the fluctuant swelling of the parotid area and lymph nodes may suggest a nonspecific autoimmune response, while recurrent exophthalmos in the absence of orbit mass may caused by venous obstruction since tumor was noted within the cavernous sinus. For Cases 2 and 3, however, a posttraumatic or postinfectious inflammatory process should be considered. But it's a pity we didn't do IgG4 test in all our cases.

The diagnosis of temporal pseudotumor is one of exclusion based on history, clinical examination, radiological tests, and especially histopathology. Clinical manifestations vary widely among cases as indicated by our and previous reports. Laboratory abnormalities include anemia, thrombocytosis, polyclonal hypergammaglobulinemia, and elevated ESR, CRP, or IgG4 [1, 28]. CT scan usually shows erosive soft-tissue mass in the middle ear and/or mastoid with occasional extensions into adjacent structures. Common MRI findings are low-signal intensity on T2-weighted images and homogeneous contrast enhancement [15, 26]. All these features, however, are not very specific. Thus, a diagnosis can only be made with histopathologic and immunohistochemical studies [1, 10], especially the latter one. Pseudotumor of the temporal bone usually occurs in isolation and involves one ear according to the literature review. To the best of our knowledge, the appearance of simultaneous lesions of the temporal bone and parotid gland has not been reported previously. Thus, Case 1 in our series may represent the first such reported case with pathological confirmation. In addition, the ipsilateral neck masses (enlarged lymph nodes) in the first two patients may also associate with the temporal bone lesions as both of them shrunk immediately after mastoid surgery, even though there was a lack of solid proof.

Treatments for temporal pseudotumor are controversial with excision, corticosteroid, radiotherapy, chemotherapy, or any combinations of the four depending on tumor location, behavior, and the condition of the patient [2–24]. Surgery is usually of the first priority. Other options have been used primarily as an adjuvant to surgery or for those with inadequate excision or recurrent disease. While Coulson et al. [16] suggest a trial of oral steroids prior to surgery, as this alone may completely resolve the patients' symptoms. The steroid regimen is often given according to doctors' experience [10, 11, 13, 16, 19], and a course of high-dose oral steroids followed by tapering is usually chosen. We cannot draw conclusions from few cases about which regimen to be the best, but we suggest further studies to reach an agreement about the type and course of the steroid therapy. 

The prognosis of IP is usually good. As far as our three patients are concerned, the outcomes are much different. In two cases treated by surgery alone, the diseases resolved completely. While the IP of the first case experienced several recurrences during followup, even though a combined modality therapy of surgery, steroid, radiotherapy and chemotherapy was chosen. This may due to the variable natural history of IP, the infiltrative and recurrent nature of focal lesion, or because of the insufficient steroid course used.

## 5. Conclusion

IP of the temporal bone is rare. It has no distinctive characteristics both clinically and radiologically, and the presenting symptoms and biological behaviors vary widely among patients. In this paper, we present three new cases of temporal pseudotumor with variable clinical manifestations and treatment outcomes. In two of them, a possible involvement of cervical lymph node and/or concomitant IP of the parotid gland were found. To the authors' knowledge, our first patient represents the only reported case so far to have a simultaneous appearance of IPs both in the temporal bone and in the parotid gland.

## Figures and Tables

**Table 1:** Clinical features of cases of temporal pseudotumor reported in the literature.

Reported cases	Age/sex	Symptoms	Location	Treatment	Outcome
Yanagihara et al. [2]	41/M	Recurrent FP	ME/facial nerve	Surg	NED
Benton et al. [3]	37/M	Ota	ME/M	Surg/Radio	NED at 1 yr
Nam et al. [4]	24/F	Ota/HL/Dizz/RP	M/dura	Surg/Radio	NED at 1 yr
Wiseman et al. [5]	4/M	FP	ME/M/facial nerve	Surg	NED
	39/M	HL	ME/M/Lab/IAC	Surg	NED
Mulder et al. [6]	38/F	HL	ME/M/Lab/IAC	Surg	NED
	50/F	HL	ME/M/Lab/IAC	Surg	Relapsed
Janicki et al. [7]	55/M	Oto/Tinn/vertigo	ME/M/dura/petrous apex	Radio	NED at 2 mo
	39/F	Ota/Oto/vertigo/HL	ME/M	Surg/steroid/IST	Relapsed at 4 mo
Schönermark [8]	49/F	Ota/Oto/HL	ME/M/Lab	Surg/steroid	NED at 30 mo
	35/F	FP/HL/Ota/Oto	ME/M/Lab	Surg/IST	NED at 2 yrs
	59/M	Ota/Oto/HL	ME/M/Lab	Surg/IST/steroid	Died of the disease
Gasparotti et al. [9]	26/M	Vertigo	M/Teg/Sig/dura	Surg	NED
Williamson et al. [10]	49/M	HL/Oto	ME/M/Teg/MCF/Lab	Surg/steroid	NED at 6 mo
Cho et al. [11]	42/F	Hea/HL/FP/diplopia/ visual dimness	M/ME/dura/tentorium/ cavernous sinus	Steroid	Remission at 3 mo
Cho et al. [12]	55/F	HL/Oto	ME/M	Surg	NED at 1 yr
Lee et al. [13]	39/M	HL/RP/diplopia	M/petrous apex/lung	Surg/steroid	NED at 1 yr
Lee et al. [14]	28/F	FP/Ota	M	Surg	NED at 3 yrs
Strasnick and Vaughan [15]	65/F	HL/Dizz/Tinn	M/Sig/MCF	Surg/Radio/steroid	NED at 8 yrs
	40/M	HL/facial pain/ ear fullness	Petrous apex/M/ME	Surg/Radio	NED at 10 yrs
Coulson et al. [16]	60/M	HL/Oto/Ota/FP	M/ME/brain parenchyma	Steroid	NED at 18 mo
Lee et al. [17]	7/F	HL/ear-fullness	ME/M	Surg	NED at 6 mo
Allona et al. [18]	28/M	Hea	Petrous apex/Sig	No	Remission at 1 yr
Ajibade et al. [19]	41/M	Ota/HL/Tinn/Oto	M/Teg/dura	Surg/steroid	Relapsed at 4 mo
Curry et al. [20]	2.5/M	Ota/fluctuating FP	ME/M/Lab	Surg/steroid	NED at 1.5 yrs
Santaolalla-Montoya et al. [21]	75/M	Hea/Oto/HL	ME/M/Petrous apex/dura	Surg/steroid	Died of the disease
Galindo et al. [22]	28/M	HL/Hea/Ota	Petrous apex/ME	No	Remission at 3 yrs
Goh et al. [23]	27/F	Ota/HL	ME/M (bilateral)	Surg/steroid	NED at 3 yrs
Vasileios et al. [24]	19/M	auricular polyp	ME/External ear	Surg	NED at 6 mo

Ota: otalgia; HL: hearing loss; Dizz: dizziness; Tinn: tinnitus; RP: retrobulbar pain; Oto: otorrhea; Hea: headache; FP: facial palsy; Exo: exophthalmos; ME: middle ear; M: mastoid; Lab: labyrinth; Teg: tegmen tympani; MCF: middle cranial fossa; Sig: sigmoid sinus; IAC: internal auditory canal; Surg: surgery; Radio: radiotherapy; IST: immunosuppressive therapy; NED: no evidence of disease at followup.
